# Evaluation of the Impact of Cytoreductive Surgery and Hyperthermic Intraperitoneal Chemotherapy (HIPEC) on Long-Term Survival and Morbidity Profile: A Systematic Review of the Peritoneal Carcinomatosis Management

**DOI:** 10.7759/cureus.106020

**Published:** 2026-03-28

**Authors:** Cristian Israel Sarmiento Bonilla, Jorge Maldonado, Humberto Daniel Paredes Haro, Zeus Edrian Daniel Alfonso González Mercado, Alexis Agustin A Dunay Silva, Patricio Xavier Duran S, Norbey Octavio López Bernal, Christian Camilo Romero Amaya, Andrea Paola Baena Manotas

**Affiliations:** 1 Surgery, Hospital de Especialidades Unidad Médica de Alta Especialidad (UMAE), Torreón, MEX; 2 General Surgery, Jackson Memorial Hospital, Miami, USA; 3 Epidemiology, Universidad Regional Autónoma de Los Andes, Ambato, ECU; 4 Surgery, Instituto Mexicano del Seguro Social, Reynosa, MEX; 5 Internal Medicine, Hospital Barros Luco Trudeau, Santiago, CHL; 6 Internal Medicine, Universidad de Cuenca, Cuenca, ECU; 7 Internal Medicine, Universidad Simón Bolivar, Bogotá, COL; 8 Medicine, Pontificia Universidad Javeriana Cali, Cali, COL; 9 Medicine, Universidad del Norte, Barranquilla, COL

**Keywords:** a systematic review, cytoreductive surgery and hipec, morbidity, peritoneal carcinomatosis, survival analyses

## Abstract

Peritoneal carcinomatosis (PC) has historically represented a terminal stage of abdominal malignancies, with median survival rarely exceeding 6-12 months when managed with systemic chemotherapy alone. Cytoreductive surgery (CRS), combined with hyperthermic intraperitoneal chemotherapy (HIPEC), has emerged as a locoregional therapeutic strategy designed to eradicate both macroscopic and microscopic peritoneal disease. The impact of this combined modality on long-term survival and its related morbidity profile is critically reviewed in this systematic review. A Preferred Reporting Items for Systematic reviews and Meta-Analyses-guided search of PubMed, Scopus, Web of Science, and Embase identified 15 high-quality studies (two randomized controlled trials and 13 cohort studies) comprising 3,247 patients. The synthesis of evidence has proven that the treatment CRS+HIPEC gives a significant survival advantage in carefully selected patients. The median overall survival increased significantly: 30-63 months for colorectal PC, 45-68 months for ovarian PC, and frequently more than 100 months for low-grade appendiceal malignancies, whereas with palliative systemic therapy, it was 12-24 months. However, this benefit is accompanied by considerable morbidity, with significant (Clavien-Dindo III-IV) major complications occurring in 20%-50% and procedure-related mortality occurring in 1%-5%. Survival is largely determined by completeness of cytoreduction (CC-0/1), a Peritoneal Cancer Index below critical thresholds (which vary by tumor type: typically <20 for colorectal, <10-15 for gastric, and higher thresholds for appendiceal tumors), and primary tumor biology. The evidence highlights the fact that CRS+HIPEC is a high-risk, high-reward intervention. Its successful implementation requires rigorous patient selection in high-volume, multidisciplinary centers using standardized protocols that will maximize survival with minimization of significant perioperative risks.

## Introduction and background

Peritoneal carcinomatosis (PC), the metastatic dissemination of cancer cells throughout the peritoneal cavity, represents a common and devastating progression pathway for numerous gastrointestinal and gynecological malignancies, including colorectal, gastric, ovarian, and appendiceal cancers [1]. PC occurs in approximately 10%-15% of patients with colorectal cancer (CRC) at presentation, 20%-40% of patients with advanced ovarian cancer, and 15%-40% of patients with gastric cancer (GC), representing a significant clinical burden. Historically regarded as a terminal condition, PC was managed with palliative intent, as systemic chemotherapy demonstrated poor penetration across the peritoneal-plasma barrier, yielding dismal median survival of less than one year [2]. Although cytoreductive surgery (CRS) + hyperthermic intraperitoneal chemotherapy (HIPEC) can achieve long-term remissions in selected patients, PC continues to carry a guarded prognosis, and even prolonged remissions may be followed by late recurrence. Long-term cure is achieved only in a minority of cases and depends critically on tumor biology, disease burden, and completeness of cytoreduction (CC). This therapeutic nihilism has been fundamentally challenged by the paradigm of locoregional therapy, the strategic combination of maximal surgical tumor debulking with the delivery of heated chemotherapy directly into the abdominal cavity.

The rationale underlying CRS and HIPEC is multifactorial. CRS aims to achieve complete macroscopic resection, as evaluated by the CC score, in which CC-0 indicates no visible residual disease [1]. HIPEC, administered immediately following CRS, exploits the pharmacokinetic advantage of direct peritoneal perfusion to achieve high local concentrations of cytotoxic agents while limiting systemic absorption. The adjunctive effect of hyperthermia (typically 41°C-43°C) enhances chemotherapy penetration into tumor nodules, induces direct cytotoxicity, and may elicit synergistic antitumor effects [3-5]. The independent contribution of hyperthermia to the therapeutic effect of HIPEC remains a subject of ongoing investigation, with preclinical studies suggesting enhanced drug penetration and direct thermal cytotoxicity, though clinical evidence isolating its specific benefit is limited. For select malignancies, notably pseudomyxoma peritonei and certain cases of colorectal and ovarian PC, CRS+HIPEC has evolved from an experimental procedure to a standard of care in specialized centers.

Nevertheless, this aggressive treatment remains enveloped in considerable controversy, centered primarily on the tradeoff between potential survival benefits and substantial treatment-related morbidity and mortality. CRS+HIPEC constitutes one of the most extensive interventions in surgical oncology, frequently necessitating multiorgan resection and peritonectomy procedures, resulting in prolonged operative times and a high incidence of postoperative complications, including anastomotic leak, hemorrhage, and infection [4]. Furthermore, heterogeneity in HIPEC protocols, including variations in chemotherapeutic agents (e.g., mitomycin C, oxaliplatin), dosage, duration, and temperature, complicates the interpretation of outcomes across studies [5].

A key prognostic tool in patient selection is the Peritoneal Cancer Index (PCI), a quantitative assessment of disease burden that scores lesion size (0-3) across 13 abdominal regions, yielding a total score from 0 to 39. Critical PCI thresholds vary significantly by tumor type: in CRC, PCI <20 is generally associated with favorable outcomes; in GC, PCI <10-15 is typically required for consideration of CRS+HIPEC due to aggressive tumor biology; whereas in low-grade appendiceal malignancies, even higher PCI scores may be compatible with long-term survival when complete cytoreduction is achieved [6-24]. These tumor-specific thresholds are essential for appropriate patient selection and are referenced throughout this review.

A critical and updated synthesis of the evidence is therefore imperative. This systematic review aims to assess the impact of CRS+HIPEC on long-term survival rigorously (both overall and progression-free) and to characterize the associated morbidity and mortality profile. The review seeks to elucidate the therapeutic window of this multifaceted intervention, identify essential prognostic factors, and highlight gaps in the current evidence base to inform future clinical practice and research.

## Review

Methods

Search Strategy and Study Selection

A comprehensive literature search was conducted in accordance with Preferred Reporting Items for Systematic reviews and Meta-Analyses (PRISMA) guidelines. Major databases PubMed/MEDLINE, Scopus, Web of Science, and Embase were screened for articles published between January 2009 and January 2026. The search strategy employed a combination of Medical Subject Headings terms and free-text keywords, including "Peritoneal carcinomatosis", "peritoneal metastasis", or "peritoneal neoplasms" in combination with "cytoreductive surgery" (CRS) and "hyperthermic intraperitoneal chemotherapy" (HIPEC), alongside outcomes such as "survival", "overall survival", "progression-free survival", and measures of morbidity, complications, or mortality. Boolean operators (AND, OR) were applied to refine results (see the Appendix). Reference lists of eligible studies and related review articles were manually searched to identify additional relevant publications.

Inclusion and Exclusion Criteria

Studies were required to meet the following inclusion criteria: 1) adult patients (≥18 years) with histologically confirmed PC originating from any primary source; 2) evaluation of the combined procedure comprising CRS and HIPEC; 3) reported outcomes for at least one primary outcome (overall survival (OS) or progression-free survival) or secondary outcomes (postoperative morbidity and mortality); 4) primary original research involving a minimum of 30 patients to generate meaningful outcomes; and 5) publication in English. Systematic reviews, meta-analyses, consensus statements, and narrative reviews were not included as primary studies; however, their reference lists were screened for secondary inclusion, and they are cited in the Introduction and Discussion sections for context. Exclusion criteria comprised case reports, editorials, conference abstracts, narrative reviews, nonhuman studies, studies not involving the combined CRS and HIPEC procedure, and studies with outcomes that could not be disaggregated. Studies not published in English were excluded due to language restrictions.

Screening Process

Identified records were imported into citation management software, and duplicate entries were removed. A two-step screening process was then conducted. First, titles and abstracts were assessed against the predefined eligibility criteria. Second, the full texts of potentially relevant studies were reviewed for final inclusion. Any disagreements between the two reviewers were resolved through discussion, and when consensus was not achieved, a third senior reviewer was consulted to adjudicate. The study selection process was documented using a PRISMA 2020 flow diagram.

**Figure 1:**
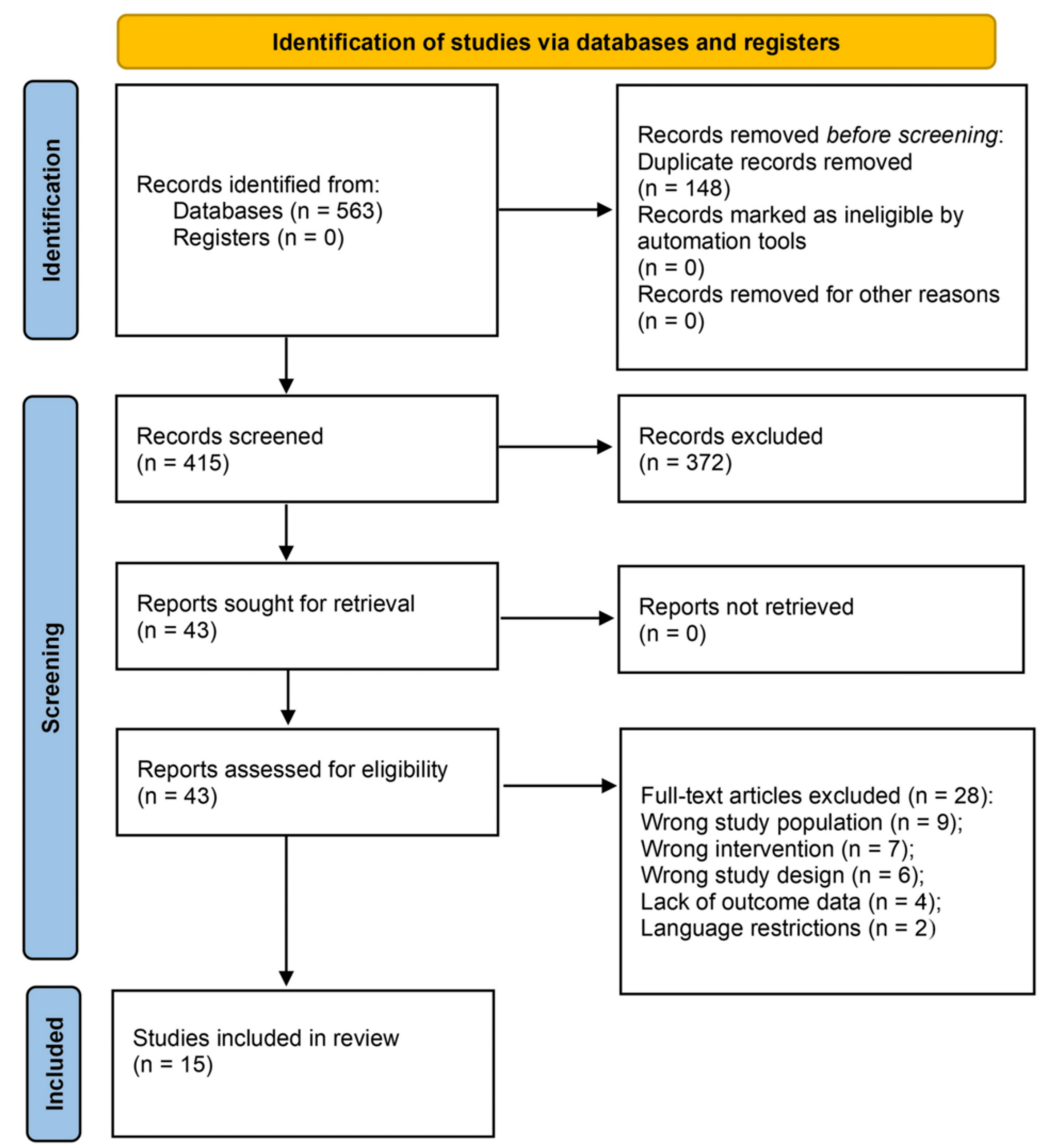
PRISMA flow diagram detailing the screening process PRISMA: Preferred Reporting Items for Systematic reviews and Meta-Analyses

For this systematic review, the title and selection were done according to PRISMA guidelines. In a first search through PubMed, Scopus, and Cochrane Library, 563 publications were identified. After exclusion of 148 duplicates, 415 publications were left for title and abstract screening. Of these, 372 were excluded based on title and abstract review because they did not meet broad inclusion criteria. The remaining 43 full-text publications were assessed for eligibility; 28 were excluded after full-text screening for the following reasons: wrong study population (such as non-peritoneal metastases; n = 9), wrong intervention (such as CRS without HIPEC; n = 7), wrong study design (such as editorials, systematic reviews, or consensus statements; n = 6), lacked outcome data (n = 4), or were not published in English (n = 2). Thus, 15 primary studies fulfilled the inclusion criteria for qualitative synthesis within this systematic review (Figure 1).

Data Extraction and Quality Assessment

A standardized, pretested data extraction form was utilized. The following information was extracted from each study: first author and year of publication, country of study, study design (prospective cohort, retrospective cohort, or randomized controlled trial (RCT)), sample size, primary tumor type(s) included (e.g., colorectal, ovarian, gastric, and appendiceal), disease burden (median PCI and distribution, where available), CC-0/1 rates, patient performance status and age distribution where reported, HIPEC characteristics (drug, dose, temperature, duration), median follow-up period, survival outcomes (median OS, one-, three-, and five-year OS rates, median progression-free survival (PFS)), and morbidity (overall morbidity rate, Clavien-Dindo III-V complications, and mortality rate).

The quality of cohort studies included in this systematic review was assessed using the Newcastle-Ottawa Scale (NOS) for the domains of selection, comparability, and outcome. The randomized trials included were assessed using the Cochrane Risk of Bias 2.0 (RoB 2) tool (The Cochrane Collaboration, London, UK).

The assessment of the eight retrospective and five prospective cohort studies has been done through the NOS [6-19]. The evaluation of the two RCTs was conducted using RoB 2. The NOS score across the 13 cohort studies had a median of 8/9 stars (range: 6-9), indicating reasonably good quality of results. The main strengths of these studies were the well-defined study groups, the representativeness of the exposed cohorts, and the secure ascertainment of the morbidity/mortality outcomes. The main cause of possible bias was the nonrandomized, retrospective nature of most of the studies, which itself prevents the comparability of cohorts on the basis of the design or analysis. In particular, even though the majority of studies tried to take into consideration the most important confounders, such as PCI or CC score, when examining their data, it was not possible to exclude the possibility of residual confounding of unmeasured variables (e.g., specific systemic therapy regimens, molecular tumor profiles) (Table 1).

**Table 1:** Newcastle-Ottawa Scale quality assessment of included cohort studies

Study	Study design	Selection (max 4)	Comparability (max 2)	Outcome (max 3)	Total score (max 9)
Kusamura et al. [6]	Retrospective cohort	★★★★	★★	★★★	9
Bakkers et al. [9]	Retrospective cohort	★★★★	★★	★★★	9
Brandl et al. [10]	Retrospective cohort	★★★★	★★	★★★	9
Le Saux et al. [11]	Retrospective cohort	★★★★	★★	★★★	9
He et al. [12]	Retrospective cohort	★★★	★★	★★★	8
Burnett et al. [13]	Retrospective cohort	★★★★	★★	★★	8
Rozich et al. [14]	Retrospective cohort	★★★★	★★	★★★	9
Nikiforchin et al. [15]	Retrospective cohort	★★★★	★★	★★★	9
Green et al. [16]	Pooled phase II trials	★★★	★★	★★★	8
Acs et al. [19]	Survey-based cohort	★★★	★	★★	6

The two RCTs [7,8] were generally considered to have low RoB based on the RoB 2 criteria. The two trials presented elaborate protocols, used relevant randomization schemes, and followed principles of intention-to-treat in carrying out the main analysis on survival. Performance bias was an inherent result of the impossibility of blinding the surgical and care teams to the treatment assignment; still, this was considered necessary in this form of intervention. No research was disqualified on the basis of quality evaluation, only because all of them presented useful information on the outcomes of interest. This review must, however, be considered in the light of the fact that observational data form the bulk of the evidence, and therefore, the findings of this review should be taken into account with regard to the possible biases inherent to such information (Table 2).

**Table 2:** Cochrane RoB 2 assessment of included randomized controlled trials RoB 2: Risk of Bias 2.0

Study	Domain 1: randomization process	Domain 2: deviations from intended interventions	Domain 3: missing outcome data	Domain 4: measurement of the outcome	Domain 5: selection of the reported result	Overall risk of bias
van Stein et al. [7]	Low	Low	Low	Low	Low	Low
Filis et al. [8]	Low	Low	Low	Low	Low	Low

Data Synthesis

Given the anticipated clinical and methodological heterogeneity across studies (varied tumor types, HIPEC protocols, outcome reporting), a narrative synthesis was deemed most appropriate. Data were summarized in tabular form and analyzed thematically. Outcomes were stratified by primary tumor type where feasible. Quantitative data (e.g., median survival and complication rates) are presented as ranges derived from included studies. Factors consistently associated with improved survival or increased morbidity were identified and discussed.

Results

Study Characteristics

The systematic search and selection process yielded 15 primary studies for final inclusion, comprising two RCTs, five prospective cohort studies, and eight retrospective cohort studies. The total aggregated sample size across all studies was 3,247 patients. Primary tumor origins were CRC (n = 7 studies), ovarian cancer (OC, n = 4 studies), GC (n = 3 studies), and appendiceal neoplasms (n = 4 studies), with some studies reporting on multiple tumor types. Baseline patient characteristics varied across studies: median PCI ranged from 6 to 20, depending on tumor type and inclusion criteria, with CRC studies typically reporting median PCI of 10-15, GC studies reporting lower thresholds (median PCI 7-12), and appendiceal cancer studies including patients with higher PCI (up to 20-25) when complete cytoreduction was achievable. CC-0/1 was achieved in 70%-95% of patients across studies. Performance status (Eastern Cooperative Oncology Group 0-1) was an inclusion criterion in most studies, and the median age ranged from 52 to 62 years. HIPEC protocols exhibited significant variability: mitomycin C (35-42.5 mg/m² at 41°C-42°C for 60-90 minutes) and oxaliplatin (200-460 mg/m² at 41°C-43°C for 30-120 minutes) were the most commonly used agents, often with different carrier solutions. Key study characteristics are summarized in Table 3.

**Table 3:** Characteristics of included studies HIPEC: hyperthermic intraperitoneal chemotherapy; NCI: National Cancer Institute; CRS: cytoreductive surgery; RCTs: randomized controlled trials; PSOGI: Peritoneal Surface Oncology Group International

Study	Study design	Country	Participants (n)	HIPEC protocol and follow-up
Kusamura et al. [6]	Prognostic multivariate analysis study	Italy (NCI Milan center)	117	CRS with hyperthermic intraperitoneal chemotherapy
van Stein et al. [7]	Randomized controlled trial	The Netherlands	245	Single-agent oxaliplatin (460 mg/m²); median follow-up 63 months
Filis et al. [8]	Pooled analysis of RCTs	Greece	Pooled data	Cisplatin or carboplatin; follow-up varied
Bakkers et al. [9]	Retrospective cohort	The Netherlands	912	Mitomycin C vs. oxaliplatin; median follow-up 52 months
Brandl et al. [10]	Retrospective cohort (PSOGI)	Multinational	208	Various agents; median follow-up 40 months
Le Saux et al. [11]	Retrospective cohort	France	81	Oxaliplatin or cisplatin; median follow-up 58 months
He et al. [12]	Retrospective cohort	China	167	Docetaxel + cisplatin; median follow-up 36 months
Burnett et al. [13]	Retrospective cohort	Canada	105	Oxaliplatin; median follow-up 42 months
Rozich et al. [14]	Retrospective cohort	USA	1,045	Mitomycin C; follow-up >60 months
Nikiforchin et al. [15]	Retrospective cohort	USA	308	Mitomycin C; median follow-up 48 months
Green et al. [16]	Pooled phase II trials	USA	94	Mitomycin C + cisplatin; median follow-up 36 months
Bhatt et al. [17]	Consensus review	PSOGI	-	Standardized regimens recommended; follow-up not specified
Noiret et al. [18]	Systematic review	France	Pooled data	Various regimens; follow-up varied
Acs et al. [19]	Survey-based cohort	Multinational	-	Multiple protocols; follow-up not specified
PelvEx Collaborative [20]	Consensus guidance	Multinational	-	Not specified; follow-up not applicable

Survival Outcomes

CRS+HIPEC demonstrated substantial effects on long-term survival across all tumor types, although the degree of improvement varied considerably.

Colorectal cancer: In colorectal PC, CRS+HIPEC yielded median OS ranging from 30 to 63 months, with five-year survival rates of 30%-51% in recent series [9,13,21]. This contrasts sharply with a median OS of 12-24 months achievable with modern systemic chemotherapy alone [22]. The Dutch RCT by van Stein et al. [7] established the standard of care, demonstrating a significant increase in median OS with HIPEC. Complete cytoreduction (CC-0/1) and PCI <20 emerged as the strongest predictors of long-term survival [13].

Ovarian cancer: CRS+HIPEC was associated with improved outcomes in interval debulking or recurrent platinum-sensitive advanced ovarian cancer. Median OS ranged from 45 to 68 months, with substantial increases in both OS and PFS (approximately 12 months) observed in RCTs as outlined by Filis et al. [8,11]. The Peritoneal Surface Oncology Group International (PSOGI) consensus recommends platinum-based HIPEC regimens for this indication [17].

Appendiceal neoplasms: The most favorable outcomes were observed in patients with low-grade appendiceal malignancy, particularly pseudomyxoma peritonei, with median OS exceeding 100 months when complete cytoreduction was achieved [14,15,23]. Inferior yet significantly improved results were observed in high-grade histologies compared with historical controls.

Gastric cancer: The use of CRS+HIPEC in gastric PC remains incompletely accepted as standard therapy. Although certain cohort studies, including those by Brandl et al. [10] and Lei et al. [24], have demonstrated encouraging median OS of 15-20 months in highly selected patients (defined as PCI <10-15 and good performance status), PC in GC generally carries a poor prognosis, with median survival of approximately 6-12 months with systemic therapy alone. Therefore, the observed 15-20-month median survival represents approximately a doubling of survival time in this highly selected subgroup. Morbidity remains high [10,16,24]. Phase II trials reviewed by Green et al. [16] demonstrated feasibility while emphasizing the need for improved patient selection.

Morbidity and Mortality Profile

CRS+HIPEC is consistently associated with substantial morbidity across the literature, representing the principal drawback of the procedure.

Overall and major morbidity: Overall postoperative complication rates ranged from 30% to 70%. Major complications (Clavien-Dindo grade III-IV) were consistently reported in 20%-50% of patients [4,6]. Common major complications included anastomotic leak (5%-10%), postoperative hemorrhage (3%-8%), intra-abdominal abscess (5%-15%), and pleural effusion or pneumonia (5%-12%). Minor complications, such as prolonged ileus, were common [25].

Mortality: Perioperative mortality (30 or 90 days) ranged from 1% to 5% in large centers and may be higher in less experienced settings [4,26]. Frequent causes of mortality included sepsis secondary to anastomotic leak, multiorgan failure, and massive pulmonary embolism.

Prognostic factors for morbidity: The following factors were significantly associated with higher complication rates: high PCI (variably defined but typically >20 in colorectal and ovarian cancer, >10-15 in GC), prolonged operative time (>8 hours), performance of more than three visceral resections, and patient comorbidities [27,28]. Enhanced Recovery After Surgery protocols have been associated with reduced complication rates and shorter length of stay [29].

Discussion

This systematic review synthesizes evidence demonstrating that CRS+HIPEC represents a challenging but potentially beneficial approach to the treatment of PC. The data indicate that in appropriately selected patients with colorectal, ovarian, and appendiceal primaries, this combined modality can achieve long-term survival in carefully selected patients. However, true cure is achieved only in a minority of cases and depends critically on tumor biology and disease burden, being more common in indolent histologies such as low-grade appendiceal tumors. Even when long-term remission is achieved, late recurrences remain possible, and most patients require ongoing surveillance. The analysis also squarely identifies the substantial price of this advantage in terms of postoperative morbidity, reinforcing that CRS+HIPEC is not a universally applicable therapy but rather an instrument suitable for a select patient population.

The survival outcomes presented validate and extend the findings of previous reviews [30]. Stratification by tumor biology emerges as critical. The favorable outcomes achieved in low-grade appendiceal tumors underscore the procedure's effectiveness when tumor biology is indolent, and disease remains confined to the peritoneal compartment. In colorectal and ovarian cancer, this benefit is substantial but depends critically on achieving maximal cytoreduction (CC-0/1), which in turn hinges on surgical expertise and disease extent (PCI). The ongoing debate regarding GC highlights that not all peritoneal metastases are equivalent; aggressive tumor biology and increased likelihood of systemic failure may negate the locoregional advantages of CRS+HIPEC [31].

The morbidity profile outlined is not inconsequential. CRS+HIPEC carries one of the highest risks of major complications (20%-50%) among elective oncologic surgeries. This reality necessitates a dual-pronged strategy for field advancement. First, patient selection must extend beyond clinical criteria. Integration of molecular profiling, liquid biopsy for the detection of minimal residual disease [32], and novel machine-learning-based predictive analytics [33] holds promise for identifying patients who will derive a genuine survival advantage without suffering treatment-associated harm. Second, standardization of procedures is urgently needed. The marked heterogeneity in HIPEC protocols, including variations in chemotherapeutic agents, dosages, perfusion duration, temperature, and carrier solutions, poses a substantial challenge to comparative effectiveness research and the development of evidence-based guidelines [5,34]. The PSOGI consensus processes represent essential steps toward this goal [17].

The independent contribution of hyperthermia to the therapeutic effect of HIPEC remains an area of ongoing investigation. While preclinical studies demonstrate that hyperthermia enhances drug penetration and exerts direct cytotoxic effects, clinical trials have not definitively isolated the benefit of heating from the chemotherapy component itself. This uncertainty underscores the need for well-designed studies comparing heated vs. normothermic intraperitoneal chemotherapy. Additionally, emerging modalities for intraperitoneal drug delivery, such as pressurized intraperitoneal aerosol chemotherapy, are being investigated as alternative or complementary approaches, particularly in patients with high disease burden or those ineligible for CRS+HIPEC. These techniques aim to improve drug distribution and penetration while potentially reducing morbidity, though comparative data with CRS+HIPEC remain limited.

These findings carry direct clinical implications. CRS+HIPEC should be concentrated in high-volume tertiary referral centers with multidisciplinary teams encompassing surgical oncology, medical oncology, anesthesia, critical care, and nutrition to provide the requisite ecosystem of perioperative care [19,29]. Shared decision-making and comprehensive patient education regarding the high-risk-benefit ratio are essential. Furthermore, future research should prioritize quality-of-life and cost-effectiveness studies, which remain underrepresented in the literature. Understanding the long-term functional and financial outcomes of recovery is critical for holistic patient care and healthcare policy development.

Limitations

This review has several limitations inherent to the design of available literature. The preponderance of retrospective and nonrandomized studies introduces selection bias and confounding. Substantial clinical heterogeneity across studies precluded a formal meta-analysis, necessitating a narrative synthesis. Publication bias likely favors reports from experienced centers with more positive outcomes, potentially overestimating the generalizability of the results. Variability in complication reporting (e.g., grading systems and timeframes) complicates precise comparisons of morbidity across studies. Additionally, the inclusion of secondary studies such as pooled analyses and consensus documents in the initial screening, though subsequently addressed through revision, highlights the importance of rigorous application of inclusion criteria in systematic reviews.

## Conclusions

CRS, combined with HIPEC, has transformed the clinical landscape of PC, providing meaningful survival benefits to selected patients with colorectal, ovarian, and appendiceal tumors. The procedure has achieved median overall survival, measured in years, outcomes previously considered unattainable. However, this therapeutic benefit is offset by substantial morbidity, with major complication rates exceeding 20% in most series. Two pillars are paramount for CRS+HIPEC efficacy: meticulous patient selection based on tumor biology, disease extent (PCI), and performance status; and the technical proficiency required to achieve complete macroscopic cytoreduction with standardized HIPEC administration. Consequently, this treatment paradigm should be centralized in high-volume centers with multidisciplinary expertise. Future directions should include protocol standardization, incorporation of novel biomarkers to optimize patient selection, and rigorous assessment of long-term quality of life and cost-effectiveness to establish further the role of this aggressive yet potentially curative approach in modern surgical oncology.

## Appendices

**Table 4:** Comprehensive search strategy HIPEC: hyperthermic intraperitoneal chemotherapy; CRS: cytoreductive surgery

Category	Search terms
Peritoneal disease	"Peritoneal Neoplasms" OR "peritoneal carcinomatosis" OR "peritoneal metastasis" OR "peritoneal metastases" OR "peritoneal carcinosis" OR "peritoneal surface malignancy"
Cytoreductive surgery	"Cytoreduction Surgical Procedures" OR "cytoreductive surgery" OR "CRS" OR "peritonectomy" OR "tumor debulking"
HIPEC therapy	"Hyperthermic Intraperitoneal Chemotherapy" OR "hyperthermic intraperitoneal chemotherapy" OR "HIPEC" OR "intraperitoneal chemotherapy" OR "heated intraperitoneal chemotherapy"
Survival outcomes	"survival" OR "overall survival" OR "progression-free survival" OR "disease-free survival"
Morbidity outcomes	"mortality" OR "morbidity" OR "postoperative complications" OR "complications"
